# Imaging Manifestations of Lung Injury During the COVID-19 Outbreak: What Have We Learned?

**DOI:** 10.5041/RMMJ.10415

**Published:** 2020-07-31

**Authors:** Anat Ilivitzki, Bar Rinnot, Luda Glozman

**Affiliations:** 1 Pediatric Radiology Unit, Department of Radiology, Rambam Health Care Campus, Haifa, Israel; 2 The Ruth & Bruce Rappaport Faculty of Medicine, Technion–Israel Institute of Technology, Haifa, Israel

**Keywords:** B-lines, COVID-19, ground-glass opacification, lung imaging, lung ultrasound, subpleural consolidation

## Abstract

Coronavirus disease-19 (COVID-19) is a pandemic infectious disease caused by a novel coronavirus. Infection can result in a wide range of clinical outcomes, from an asymptomatic condition to severe bilateral pneumonia and life-threatening conditions. Diagnosis is based on the combination of a history of exposure, clinical presentation, and real-time polymerase chain reaction (RT-PCR) assays. In endemic areas, imaging tests including computed tomography (CT), chest X-ray (CXR), and ultrasound (US) have been included in the diagnostic workup. Multiple and peripheral areas of parenchymal injury is the hallmark of COVID-19 lung infection, seen as ground-glass opacification and consolidation on CT, as hazy opacities on CXR, and as multiple B-lines and subpleural consolidations on US. Of these modalities, CT has the best sensitivity and specificity, while CXR has moderate sensitivity and unknown specificity. Both CT and CXR involve ionizing radiation, increase the risk of cross-infection, and require a long sterilization time. Ultrasound is the only modality used by clinicians. Early reports have shown promising results, comparable to CT. With high availability, the lowest risk of cross-infection, and a rapid sterilization process, US may potentially become the primary imaging tool for COVID-19 pulmonary injury. Lung US training programs are needed to provide clinicians with the ability to better implement this technique.

## INTRODUCTION

Coronavirus disease-19 (COVID-19) is a pandemic infectious disease caused by the novel coronavirus, severe acute respiratory syndrome coronavirus 2 (SARS-CoV-2). Ribonucleic acid (RNA) viruses known to have caused severe acute respiratory syndrome (SARS) and Middle East respiratory syndrome (MERS) are in the same virus family.^1^ The COVID-19 outbreak started in Wuhan, China, in December 2019 and rapidly spread worldwide; it was officially declared a pandemic by the World Health Organization (WHO) in March, 2020.^2^

Infection presents with a wide range of clinical outcomes, from asymptomatic to severe bilateral pneumonia and life-threating conditions. Having an incubation period in the range of 3–14 days, its dominant clinical presentation is high temperature and cough.^3^ Diagnosis is based on a combination of parameters, including the patient’s exposure history, clinical presentation, and real-time polymerase chain reaction (RT-PCR) assay from oropharyngeal or nasopharyngeal swab specimens, bronchoalveolar lavage, or tracheal aspirate, and classic findings on imaging tests including computed tomography (CT), chest X-ray (CXR), and ultrasound (US).^4^

With no known treatments and in the absence of a vaccination, the best method for controlling a new pandemic is early and accurate diagnosis of infection and isolation of patients from the healthy population. With the outbreak of the COVID-19 pandemic, RT-PCR diagnosis was available quite early; however, its total positive rate from throat swabs was quite low at initial presentation.^5^ The low sensitivity of RT-PCR indicates that a large proportion of the infected patients will not be identified, and these patients will not be treated in timely manner. Given the highly contagious nature of COVID-19, these patients constitute a high risk for its further spread. Furthermore, the short supply of PCR testing kits in endemic areas has made triage of suspected patients quite difficult.^5^

Coronaviruses are enveloped, pleomorphic, or spherical particles, 150–160 nm in size. Inhalation of infectious aerosols is the classic mode of transmission. These virus particles can be inhaled into the airway and lungs, reaching even the alveoli. This may explain why the viral pneumonia lesions are primarily in the subpleural areas. COVID-19 generally begins in the terminal alveoli, which are close to the pleura.^6^

Chest X-ray and chest CT are routinely used in diagnosis of acute lung infection and injury. In recent years, lung US has emerged as a valuable tool in assessing acute lung disease, used mainly by clinicians. All three modalities were used during the early outbreak in China and later on in Italy and other countries, for both diagnosis and monitoring of COVID-19 patients. This short review looks at the typical radiographic features of COVID-19 using these modalities, and discusses the advantages and shortcomings of each one, in light of this highly infectious and new disease.

## IMAGING STRATEGIES

In mainland China, CT was often a first-line investigation for COVID-19, and some hospitals dedicated specific CT scanners for examining suspected COVID-19 patients only.^7^ This strategy may be logical for an endemic population in which the main load of hospitalized patients is suspected or diagnosed with COVID-19; however, in a cosmopolitan region, it poses great logistic difficulty on the radiology department and presents a potential hazard of cross-infecting personnel as well as other patients. For these reasons, the American College of Radiology suggested using portable CXR as the first imaging tool,^8^ and both Italian and British hospitals shifted to that direction.^7^,^9^ Israel’s National Center for Information and Knowledge in the Battle Against the Coronavirus has not recommended the use of imaging, including CT, for diagnosing COVID-19, but rather has relied on laboratory diagnoses,^10^ with imaging reserved for monitoring of the disease in confirmed patients.

### Chest X-ray

**Figure 1:**
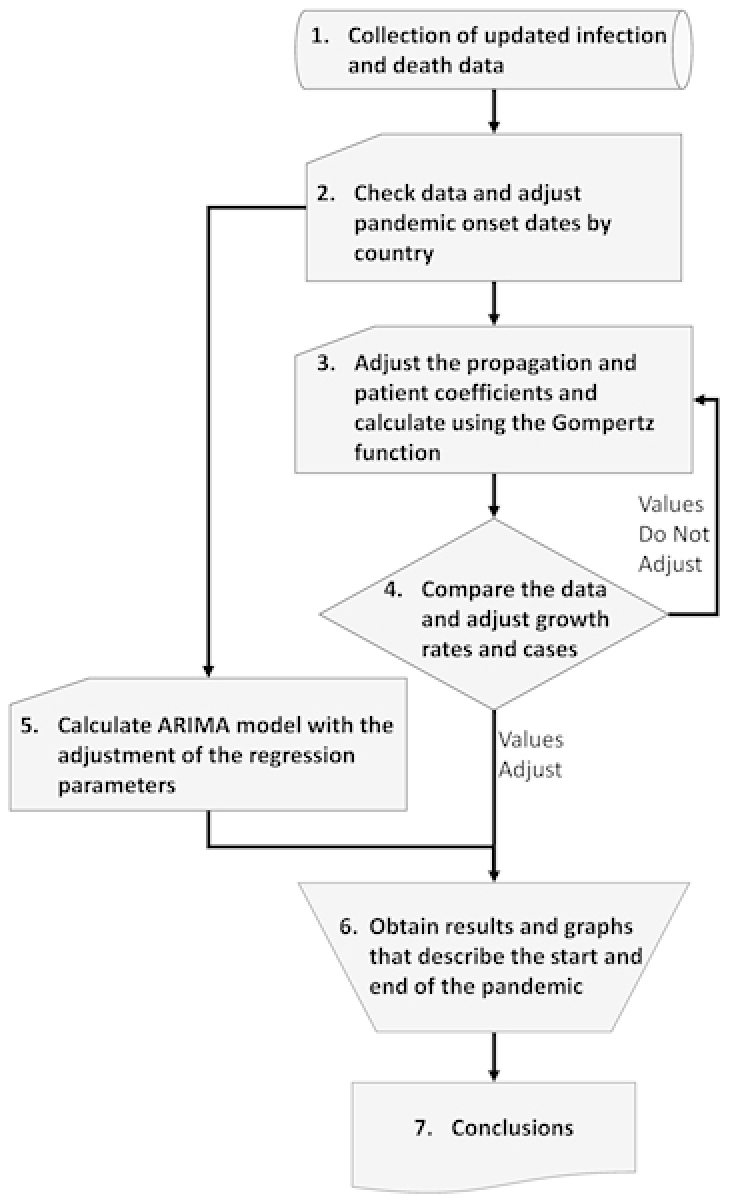
Chest X-ray (CXR) of a Confirmed COVID-19 Patient in the Sitting Position Red arrows indicate peripheral hazy lung opacities; * indicates lung consolidation in the right upper lobe; the open red arrow indicates an external device.

Chest X-rays are an effective bedside modality through the use of portable devices. The classic CXR findings in COVID-19 are areas with increased hazy opacities and/or bilateral consolidations, usually with distribution in the lower lobes.^10^–^12^ Pleural effusion is uncommon (Figure 1). The diagnostic sensitivity of CXR in the early stage of the disease is about 69%,^7^ although the specificity is unknown.

### Computed Tomography

**Figure 2:**
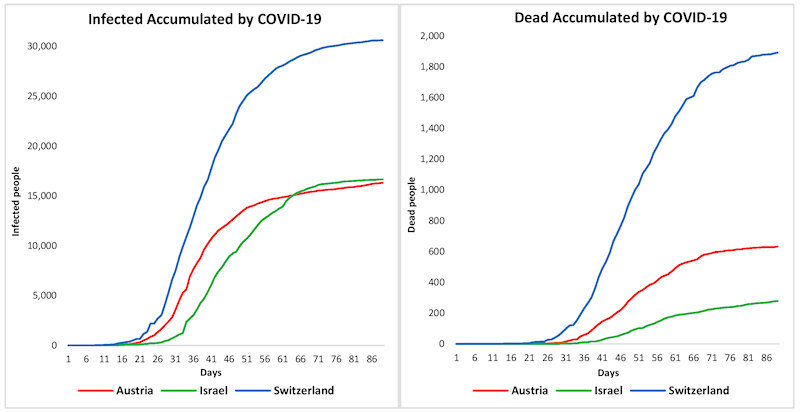
Chest Computed Tomography (CT) of a Confirmed COVID-19 Patient No contrast injected; adjusted to lung window level. Multiple, bilateral small subpleural consolidations (red arrows).

The CT findings in COVID-19 are well documented. Ground-glass opacification (GGO; defined as hazy increased lung attenuation with preservation of bronchial and vascular margins) and consolidative pulmonary opacities (defined as opacification with obscuration of margins of vessels and airway walls), often with a bilateral and peripheral lung distribution, are the hallmarks of COVID-19 patients’ chest CT images (Figure 2).^13^,^14^

**Figure 3:**
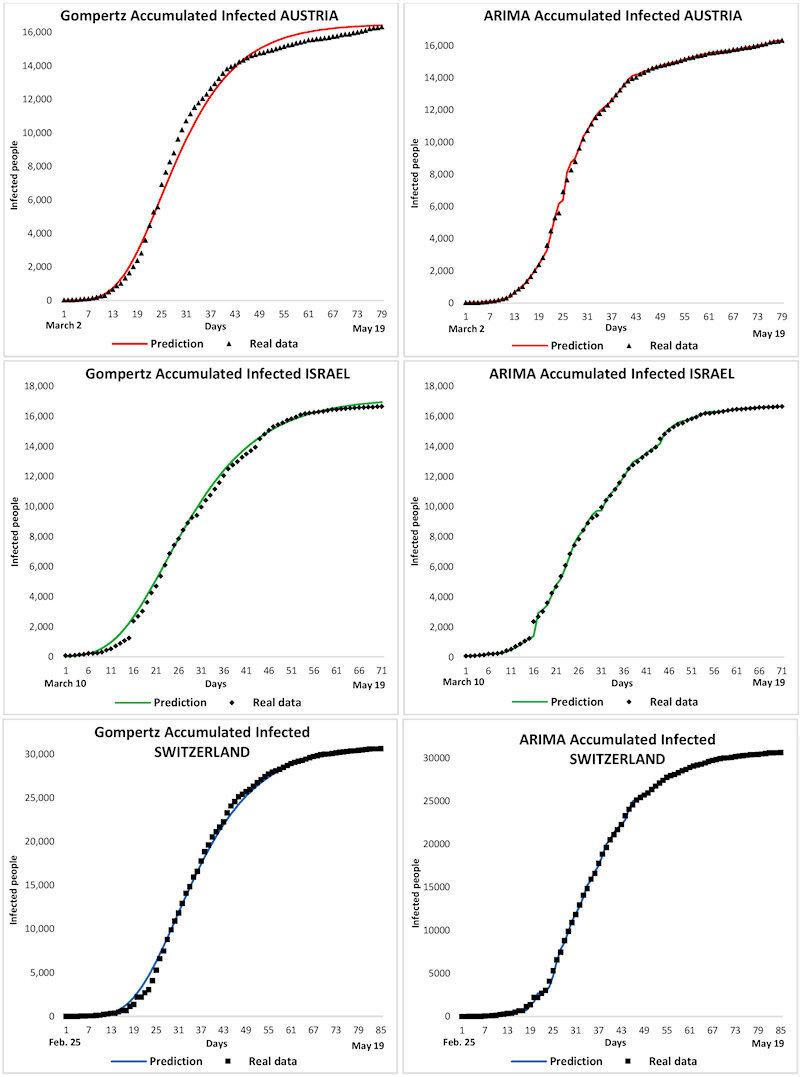
Chest CT Showing the Temporal Stages of COVID-19 Infection (Findings Indicated by Red Frame) **A:** Early phase (days 0–4)-small peripheral ground-glass opacity (GGO). **B:** Intermediate stage (days 5–8)-increased number of peripheral GGOs, and creation of “crazy paving pattern.” **C:** Intermediate stage (days 9–13)-consolidations with air bronchogram. **D:** Late phase, recovery (day 14 and onwards)-resolution of consolidation, appearance of peripheral fibrous linear stripes.

Bernheim et al. correlated the CT findings of 94 confirmed COVID-19 patients (based on positive RT-PCR) with the infection duration (early phase: days 0–2; intermediate phase at days 2–5; and late phase: days 6–12). During the first two days of the disease, 56% of patients had a normal CT as compared to 4% in the late phase. Early findings showed a bilateral distribution of small GGOs, mainly peripherally located. Over time, opacifications and consolidations increased, additional patterns such as crazy paving, a reversed halo sign, and linear opacities were noted (Figure 3), and the peripheral predilection and multilobular distribution became more apparent.^14^ Jin et al. recognized five temporal stages classified as ultra-early, early, rapid progression, consolidation, and dissipation stages. In the dissipation stage, lesions may be reduced in number and extension, with small ill-defined interlobular septal thickening remaining.^15^ These findings were in concordance with other publications, summarized in a review by Lomoro et al.^12^

The role of CT for COVID-19 diagnosis merits exploration. In endemic areas in China, due to the shortcomings of RT-PCR, CT was used for early disease detection.^7^ Ai et al. showed that CT has greater sensitivity than RT-PCR for COVID-19 diagnosis in a highly endemic area and high pre-test probability for disease.^16^ Bernheim, on the other hand, showed that during the first few days of infection more than half of the patients had a normal CT, and concluded that CT had limited sensitivity and a negative predictive value early after symptom onset, and that it was therefore not a reliable standalone tool for ruling out COVID-19 infection.^14^ Lomoro et al. showed normal CT studies for almost 5% of confirmed COVID-19 patients.^12^

Another important question regarding CT is whether or not it can differentiate between COVID-19 and other viral pneumonias. The CT findings in different virus families have been well documented, but an overlap of imaging findings and atypical presentation might complicate their interpretation.^17^ In addition, other processes such as influenza pneumonia and organizing pneumonia may manifest as bilateral peripheral GGOs.^18^ Bai et al. retrospectively compared the CT findings in COVID-19 to other viral pneumonias. They found a tendency toward peripheral rather than central pathology, mainly GGOs, fine reticular opacities, vascular thickening, with a lower frequency of pleural effusion and lymphadenopathy in COVID-19 as compared to other viral pneumonias. Radiologists using CT to differentiate between COVID-19 and non-COVID-19 pneumonia did so with moderate sensitivity (80.4%) and high specificity (96.6%), thus CT appears to be better suited to ruling out COVID-19.^19^

### Ultrasound

Lung and chest US are already being used by intensive care physicians as a bedside tool for evaluating a variety of pathologies including pneumothorax, pleural effusion, acute dyspnea, pulmonary edema, pulmonary embolism, pneumonia, interstitial processes, and for patients on mechanical ventilatory support.^20^ In the early days of the COVID-19 outbreak, clinicians were originally the ones who performed lung US on infected patients and as a result mastered the technology; they subsequently published their findings.^21^,^22^

Any recent-generation US device can be used for lung exams: premium devices, small portable ones, and even wireless US devices are all adequate for lung and thorax imaging. Different probes are used, mainly curvilinear probes with an abdominal preset for deeper and larger lung field coverage, and linear probes for better visualization of the specific superficial pathology. Phased array probes designed for cardiac imaging may also be used.^20^ The operator should be well trained and familiar with both the machine and the technique.^23^ The operator’s goal is to cover as much as possible of the lungs in a systematic, quick, and efficient manner. For reproducibility and uniformity of reporting, the lungs are scanned at fixed points. Most of the COVID-19 publications relating to lung US used a focused 12-area approach. The thorax was scanned bilaterally (Right 1–6 and Left 1–6) at the bedside: anterior superior and inferior, lateral superior and inferior, posterior superior and inferior.^21^–^26^

Any lung pathology that extends to the pleural surface may be visualized with US. However, US cannot detect lesions deep within the lung, since the aerated lung blocks its transmission.

**Figure 4:**
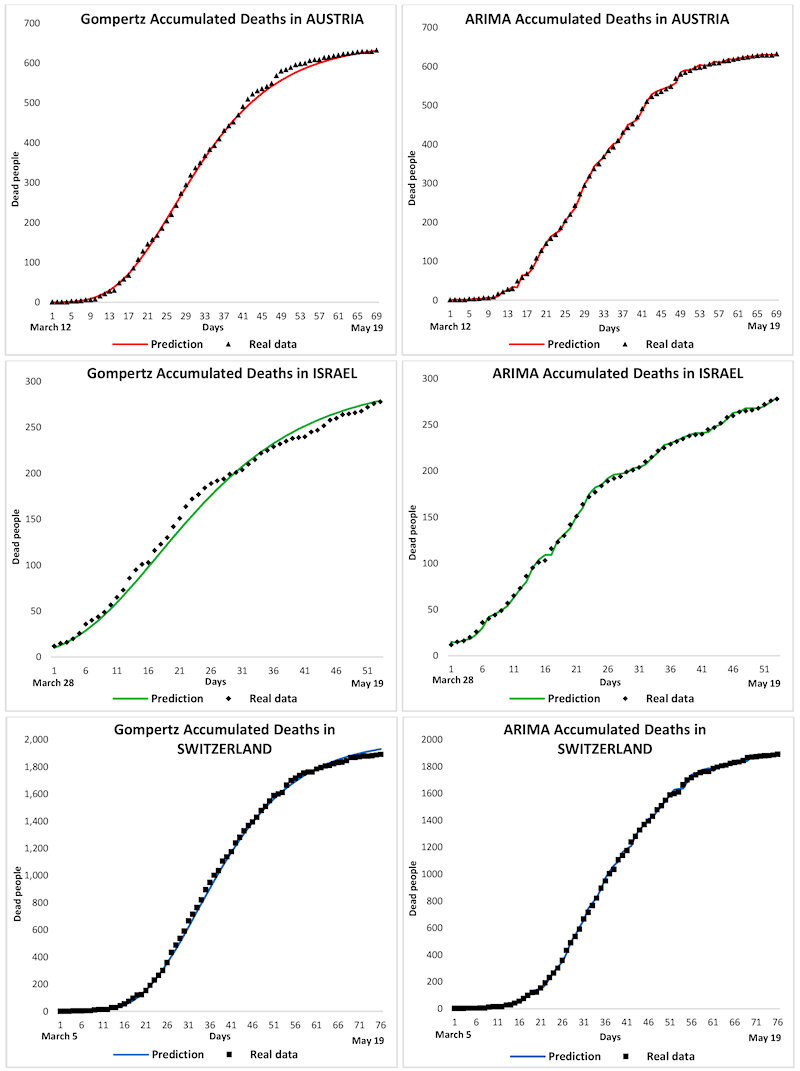
US of the Normal Lung The chest wall is seen superficially, with subcutaneous tissues and ribs (*). The normal pleura adjacent and posterior to the chest wall (red arrow), and the aerated lung seen hypoechoic with A-lines (yellow arrows).

Normal chest sonography will show the detailed chest wall anatomy including the skin, subcutaneous tissue, muscular layer, and ribs. The pleura slides during respiration and is seen as a sharp continuous white line, posterior and adjacent to the chest wall. In addition to blocking US transmission, the aerated lung is hypoechoeic and homogenic. A reverberation artifact, generated due to the interphase between the superficial layers and the air in the lungs, creates what are known as white A-lines. These A-lines appear in constant spacing, parallel to the pleural line and to each other, and fade gradually with depth (Figure 4).

In all lung pathologies, the A-line artifacts are lost and a different artifact appears, as the in B-lines, which are generated when the interstitium or small number of subpleural alveoli fill with fluid. The interphase between the aerated areas and the fluid-filled areas create the B-line artifact. If present, these longitudinal B-lines begin at the pleural line and run without fading to the deeper field. Their sharpness, number, distribution, and location differ between lung pathologies and constitute clues to diagnosis. Regardless of the specific pathology, the decay of B-lines and the reappearance of A-lines are considered to be reliable signs of lung recovery.^24^

Small subpleural consolidations are seen in US as hypoechoic areas with or without air bronchogram and local loss of the pleural line. A proper segmental to lobar consolidation with complete loss of the aerated lung parenchyma is visualized as a solid tissue, with a strong resemblance to the liver parenchyma, known as hepatization of the lung. A dynamic air bronchogram is seen within the consolidated lung.

**Figure 5:**
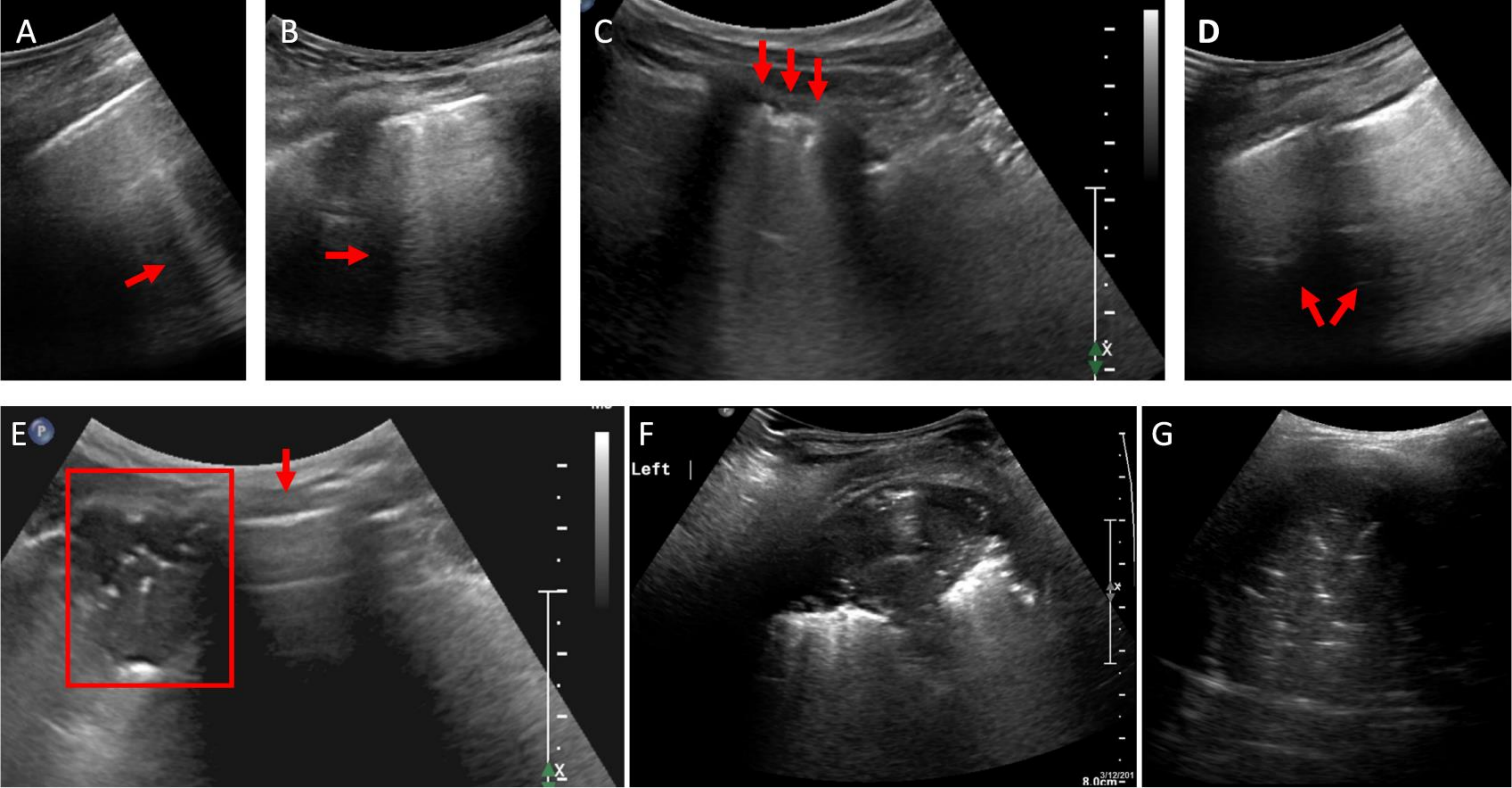
Ultrasound of a COVID-19 Lung Injury **A:** Blurred edge B-line (red arrow). **B:** Multiple blurred edge B-lines (red arrow). **C:** Confluent blurred edge B-lines (red arrows) and thick irregular pleural line. **D:** Diffused B-lines “white lung” sign (red arrows). Notice the thickened pleural line. **E:** Lung with a small subpleural consolidation (within red frame) next to a normal, aerated lung with A-lines (red arrow). **F:** Subpleural consolidation with air bronchogram. **G:** Hepatization of the lung in segmental pneumonia with large consolidation and air bronchogram.

Only a few publications, mainly case reports or small series, have described the sonographic findings in COVID-19. Classical findings include an irregular pleural line with small subpleural consolidations, disappearance of the normal A-lines, and the appearance of multiple B-lines. The B-lines have areas that are fused (waterfall sign) or diffused (white lung sign). The B-lines noted in COVID-19 are more likely to be fused, fixed, and with blurred edges than those appearing with cardiogenic pulmonary edema. Also noted are small subpleural multifocal, non-translobar, and translobar consolidations in a variety of patterns, with occasional mobile air bronchograms. Pleural effusion in COVID-19 is uncommon, and the lesions are mostly located in the posterior fields of both lungs. Spared areas are present bilaterally, mixed with pathological areas (Figure 5). With COVID-19 recovery, the A-lines reappear, replacing the B-lines.^22^

The use of lung US to evaluate several respiratory conditions has been widely documented.^20^ Lung US provides results similar to chest CT and superior to standard chest radiography for evaluation of pneumonia and/or adult respiratory distress syndrome (ARDS).^20^ Several studies have noted a good correlation between lung US and CT for COVID-19.^12^,^21^,^22^,^25^,^26^ In addition, Yang et al. showed an improved sensitivity of lung US compared to CT in 29 patients with COVID-19 pneumonia.^26^

Another advantage of lung US is the ability of clinicians to perform this procedure at the bedside. In the presence of a highly contagious pathogen such as COVID-19, the use of a single dedicated bedside US device reduces the chance of cross-infection. Since no ionizing radiation is involved, the study may be repeated as clinically needed. The 12-area method allows for precise reporting and proper follow-up. Sterilization of portable US devices, especially the smaller or wireless machines, is considerably easier and faster as compared to a CT machine. Finally, portable US machines are relatively inexpensive and can be purchased for any hospital department as well as any community clinic responsible for treating COVID-19 patients outside of the hospital setting.

## CONCLUSIONS

In general, there is a strong consensus among healthcare officials in the USA, Europe, and Israel that imaging is not the first tool of choice for diagnosis of COVID-19. Imaging is, however, justified when clinically needed in RT-PCR-confirmed COVID-19 patients. Computed tomography has been the most studied imaging modality in the COVID-19 out-break, followed by CXR and US. Although CT has the best sensitivity and specificity, it involves ionizing radiation, increases the chances of cross-infection inside the hospital, and its lengthy sterilization process makes the device unusable for long periods of time. Furthermore, for patients treated in the community, CT has low availability.

Chest X-rays have moderate sensitivity and unknown specificity. The need for portable machines operated by specialized technicians increases the risk of cross-infection.

Ultrasound, on the other hand, is the only modality used by clinicians. Its use in critically ill patients is wide-spread. In the hands of trained clinicians, US has provided promising results, comparable to CT, according to early reports in the COVID-19 outbreak. The advantages of US during a highly infectious outbreak include high availability, low risk of cross-infections, and a rapid sterilization process, in comparison to the other two modalities. In view of these observations, and especially during this highly contagious pandemic, hospitals and healthcare clinics should consider implementing lung US operated in the clinic as part of their daily follow-up of infected patients and as a tool for monitoring disease severity. Computed tomography should be added only for selected patients, as clinically needed.

To that end, there is a need for training programs for clinicians in lung US in order to better utilize the modality and improve sensitivity and specificity.

## Abbreviations

COVID-19: coronavirus disease-19
CT: computed tomography
CXR: chest X-ray
GGO: ground-glass opacification
MERS: Middle East respiratory syndrome
RNA: ribonucleic acid
RT-PCR: real-time polymerase chain reaction
SARS: severe acute respiratory syndrome; SARS-CoV-2, severe acute respiratory syndrome coronavirus; US, ultrasound; WHO, World Health Organization.
SARS-CoV-2: severe acute respiratory syndrome coronavirus; US, ultrasound; WHO, World Health Organization.
US: ultrasound
WHO: World Health Organization

## References

[1] Rothan HA, Byrareddy SN (2020). The epidemiology and pathogenesis of coronavirus disease (COVID-19) outbreak. J Autoimmun.

[2] World Health Organization Rolling updates on coronavirus disease (COVID-19).

[3] Guan W-J, Ni Z-Y, Hu Y (2020). Clinical characteristics of coronavirus disease 2019 in China. N Engl J Med.

[4] Zu ZY, Jiang MD, XU PP (2020). Coronavirus disease 2019 (COVID-19): a perspective from China. Radiology.

[5] Fang Y, Zhang H, Xie J (2020). Sensitivity of Chest CT for COVID-19: comparison to RT-PCR. Radiology.

[6] Kannan S, Ali PSS, Sheeza A, Hemalatha K (2020). COVID-19 (novel coronavirus 2019) - recent trends. Eur Rev Med Pharmacol Sci.

[7] Wong HYF, Lam HYS, Fong AH (2019). Frequency and distribution of chest radiographic findings in COVID-19 positive patients. Radiology.

[8] American College of Radiology ACR Recommendations for the use of Chest Radiography and Computed Tomography (CT) for Suspected COVID-19 Infection.

[9] Rouger M (2020). Imaging the coronavirus disease COVID-19.

[10] Chen N, Zhou M, Dong X (2020). Epidemiological and clinical characteristics of 99 cases of 2019 novel coronavirus pneumonia in Wuhan, China: a descriptive study. Lancet.

[11] Ng M-Y, Lee EYP, Yang J (2020). Imaging profile of the COVID-19 infection: radiologic findings and literature review. Radiol Cardiothorac Imaging.

[12] Lomoro P, Verde F, Zerboni F (2020). COVID-19 pneumonia manifestations at the admission on chest ultrasound, radiographs, and CT: single-center study and comprehensive radiologic literature review. Eur J Radiol Open.

[13] Hansell DM, Bankier AA, MacMahon H, McLoud TC, Müller NL, Remy J (2008). Fleischner Society: glossary of terms for thoracic imaging. Radiology.

[14] Bernheim A, Mei X, Huang M (2020). Chest CT findings in coronavirus disease-19 (COVID-19): relationship to duration of infection. Radiology.

[15] Jin Y-H, Ci L, Cheng Z-S (2020). A rapid advice guideline for the diagnosis and treatment of 2019 novel coronavirus (2019-nCoV) infected pneumonia (standard version). Mil Med Res.

[16] Ai T, Yang Z, Hou H (2020). Correlation of chest CT and RT-PCR testing in coronavirus disease 2019 (COVID-19) in China: a report of 1014 cases. Radiology.

[17] Koo HJ, Lim S, Choe J, Choi S-H, Sung H, Do KH (2018). Radiographic and CT features of viral pneumonia. Radiographics.

[18] Obadina ET, Torrealba JM, Kanne JP (2013). Acute pulmonary injury: high-resolution CT and histopathological spectrum. Br J Radiol.

[19] Bai HX, Hsieh B, Xiong Z (2020). Performance of radiologists in differentiating COVID-19 from viral pneumonia on chest CT. Radiology.

[20] Mayo PH, Copetti R, Feller-Kopman D (2019). Thoracic ultrasonography: a narrative review. Intensive Care Med.

[21] Buonsenso D, Piano A, Raffaelli F, Bonadia N, de Gaetano Donati K, Franceschi F (2020). Point-of-care lung ultrasound findings in novel coronavirus disease-19 pnemoniae: a case report and potential applications during COVID-19 outbreak. Eur Rev Med Pharmacol Sci.

[22] Peng QY, Wang XT, Zhang LN, Chinese Critical Care Ultrasound Study Group (CCUSG) (2020). Findings of lung ultrasonography of novel corona virus pneumonia during the 2019–2020 epidemic. Intensive Care Med.

[23] Expert Round Table on Ultrasound in ICU (2011). International expert statement on training standards for critical care ultrasonography. Intensive Care Med.

[24] Mojoli F, Bouhemad B, Mongodi S, Lichtenstein D (2019). Lung ultrasound for critically ill patients. Am J Respir Crit Care Med.

[25] Poggiali E, Dacrema A, Bastoni D (2020). Can lung US help critical care clinicians in the early diagnosis of novel coronavirus (COVID-19) pneumonia?. Radiology.

[26] Yang Y, Huang Y, Gao F, Yuan L, Wang Z (2020). Lung ultrasonography versus chest CT in COVID-19 pneumonia: a two-centered retrospective comparison study from China. Intensive Care Med.

